# Multifaceted *Beauveria bassiana* and Other Insect-Related Fungi

**DOI:** 10.3390/jof10020142

**Published:** 2024-02-09

**Authors:** Nicolás Pedrini, Éverton K. K. Fernandes, Ivan M. Dubovskiy

**Affiliations:** 1 Biochemistry Research Institute of La Plata (INIBIOLP), CCT La Plata Council of Scientific and Technical Research (CONICET), National University of La Plata (UNLP), Calles 60 y 120, La Plata 1900, Argentina; 2 Department of Biosciences and Technology, Institute of Tropical Pathology and Public Health, Federal University of Goiás, Goiânia 74690-900, Brazil; 3 Laboratory of Biological Plant Protection and Biotechnology, Novosibirsk State Agrarian University, Dobrolubova Str. 160, 630039 Novosibirsk, Russia; 4 Siberian Federal Scientific Centre of Agro-BioTechnologies of the Russian Academy of Sciences, 630501 Krasnoobsk, Russia

## 1. Introduction

Since Agostino Bassi first isolated the fungal pathogenic agent of the white muscardine in insects (later named *Beauveria bassiana* in his honor), and Ilya Mechnikov cultivated *Metarhizium anisopliae* as a first approach to use fungi as pest control agents, many other entomopathogenic fungi have been studied over the last two centuries [1,2].

There is evidence of a several-million-year coevolutionary history between invertebrate-pathogenic fungi and their hosts. Fungus–insect interactions are known to drive pathogenic cycles that usually culminate in killing the hosts; however, these fungi are also facultative saprophytes in the soil and/or the rhizosphere [3,4]. They can also develop endophytic relationships with plants, conferring protection to the host plant from the insects that feed on them [5]. In addition to invertebrate pathology, *B. bassiana* also has diverse applications in a range of other disciplines, including as an important whole-cell eukaryotic biocatalyst, and together with other entomopathogenic fungi, remains a reservoir for the discovery of numerous secondary metabolites with bioactive functions [6].

These topics were addressed in two Special Issues, which have captured a diversity of studies that focus on biological, molecular, and biotechnological aspects of the interaction between insect-related fungi and their wide range of hosts, including arthropods and plants, as well as on the expression of secondary metabolites, and other aspects regarding their catalyst role in biotransformation and bioremediation. A total of 11 original articles and one review article were published in the Special Issues “Multifaceted *Beauveria bassiana* and Other Insect-Related Fungi 1.0” (9 articles) and “Multifaceted *Beauveria bassiana* and Other Insect-Related Fungi 2.0” (3 articles). We briefly summarize them in the following paragraphs and encourage readers to explore them fully.

## 2. An Overview of Published Articles

The article by Amobonye et al. (contribution 1) highlights a little-studied aspect of *B. bassiana*, i.e., using whole fungal cells to produce industrially important biocatalysts. These authors purified and characterized a fungal xylanase using biochemical, spectrometric, and microscopic techniques. They showed that this enzyme is important in deinking wastepaper through enzymatic disassociation of the fiber-ink bonds. This is the first report on the characterization of a carbohydrase from *B. bassiana*, and the knowledge of this research might benefit the paper and pulp industry.

Using a gene deletion approach, three articles showed that specific genes of *B. bassiana* are involved in fungal development, metabolism, and virulence, among other traits. In this regard, Cai et al. (contributions 2) delve into the role of *BbSpt10*, a histone acetyltransferase from the GNAT superfamily protein, in cell cycle development and hyphal septation patterns. Targeted gene knockout of *BbSpt10* resulted in impaired asexual development and morphogenesis, reduced abilities to utilize various carbon and nitrogen sources, reduced tolerance to heat, fungicides, DNA damage stress, and attenuated virulence. Furthermore, comparative transcriptomics of wild-type and *ΔBbSpt10* cells revealed the differential expression of 373 genes, including 153 downregulated and 220 upregulated genes. Among the former, those involved in amino acid metabolism, cellular transportation, cell type differentiation, and virulence stand out. At the same time, the upregulated genes were enriched in carbon/nitrogen metabolism, lipid metabolism, DNA process and cell rescue, defense, and virulence. Mohamed et al. (contribution 3) showed that the deletion of *BbCre1*, a carbon catabolite repressor A from *B. bassiana*, plays a crucial role in nutrient utilization in both integument and hemocoel of insect hosts. Contrary to the study previously mentioned, most differentially expressed genes (1117 out of 1881) were downregulated in *ΔBbCre1* versus wild-type cells, leading to substantial repression of many enriched function terms and pathways, particularly those involved in carbon and nitrogen metabolisms, cuticle degradation, antioxidant response, cellular transport, and homeostasis. Finally, a second study by Cai et al. (contribution 4) focuses on *BbSirT2*. This sirtuin-class III histone deacetylase mediates fungal stress and development since *ΔBbSirT2* cells showed alterations in hyphal septation and produced morphologically aberrant conidia. Comparative transcriptomics (*ΔBbSirT2* versus wild-type cells) indicated the differential expression of 1148 genes in the former.

In contribution 5, Corrêa et al. investigated the immune response of ticks by evaluating dopamine’s effects on hemocytes and the survival of ticks inoculated with *M. anisopliae* blastospores. Exogenous dopamine increased the survival of fungus-treated *Rhipicephalus microplus* and increased the number of circulating hemocytes in ticks treated (24 h after inoculation) or not treated with fungi. Dopamine did not change the phagocytic index of tick hemocytes after 2 h. In addition, phenoloxidase activity in the hemolymph of ticks injected with both dopamine and *M. anisopliae* or exclusively with dopamine was higher than in untreated ticks or ticks inoculated with the fungus only (72 h after inoculation). Dopamine was detected in the hemocytes of *R. microplus* under physiological conditions, indicating that these cells produce dopamine naturally.

Two articles focus on screening, prospecting, and molecular characterization of entomopathogenic fungi from soils to find potential biocontrol agents for integrated pest management programs. Contribution 6 used the well-known wax moth *Galleria mellonella* entrapment method to isolate entomopathogenic fungi from the soils of the Nile Delta and explored their pathogenicity against the cotton leafworm *Spodoptera litura* and the mealworm beetle *Tenebrio molitor*. The study by Al Khoury et al. (contribution 7) characterized entomopathogenic fungi of the genus *Beauveria* in soils of Lebanon cedar forests. These authors used a combination of fungal bioexploration methods, including insect bait and selective media, to isolate a total of 249 fungi, including two novel indigenous species: *Beauveria tannourinensis* sp. nov. and *Beauveria ehdenensis* sp. nov. 

The role of entomopathogenic fungi as endophytes in plants is an increasingly studied field, specifically on their activity as biostimulants of crops and as bioinsecticides of insect pests that feed on them [7]. In this regard, the article by Vianna et al. (contribution 8) screens the endophytic capacity of 24 isolates of entomopathogenic fungi in tobacco plants and the effect on leaf consumption by its pest, the cucurbit beetle *Diabrotica speciosa*. Two isolates of *B. bassiana* exhibited the best endophytic capacity up to 28 days post-inoculation by foliar spraying; however, insect feeding behavior was similar on both colonized and non-colonized leaves.

The review article (contribution 9) summarizes the information available from transcriptomics and quantitative PCR studies related to the expression of secondary metabolite genes of *B. bassiana* inside different insects as the infection progresses, as well as for the host immune response, to help understand the mechanisms that these fungal toxins trigger as virulence factors, antimicrobials, or immunosuppressives within the context of a fungus–insect interaction.

Entomopathogenic fungi are dimorphic; transitions between penetrating germ tubes to hyphal bodies at the beginning of the infection process are triggered by the high osmotic pressure present in insect hemocoel [3], and transitions from hyphal bodies to mycelia in late infection are a consequence of a quorum-sensing system [8]. The article from Ramírez-Ordorica et al. (contribution 10) explored the role of *B. bassiana* volatiles as quorum sensing-like signals during hyphal bodies to mycelial transition. These authors outlined the fungal volatile fingerprint through the use of gas chromatography coupled to mass spectrometry and found that 3-methylbutanol retarded such transition.

Entomopathogenic fungi can be used to control insect-borne diseases, but some questions regarding their practical applications remain unclear [3]. The study of Gomes et al. (contribution 11) uncovered the virulence of *M. anisopliae*, both conidia and blastospores, against the mosquito *Aedes aegypti* under totally shaded or partially shaded conditions. The authors highlight that *M. anisopliae* blastospores were more virulent than conidia to mosquito larvae. Blastospores remained active under field conditions, but solar radiation caused delayed mortality of insects.

Although effective against arthropods, entomopathogenic fungi may be limited by the chemical composition of the arthropods’ cuticle. Ribeiro-Silva et al. (contribution 12) reported that cuticular lipid compounds extracted directly from *Dermacentor nitens* or *Amblyomma sculptum* inhibited the mycelial growth of *M. robertsii*. In contrast, the total cuticular extract from *Rhipicephalus microplus* or *Rhipicephalus sanguineus* sensu lato did not inhibit the growth of either *M. robertsii* or *B. bassiana*. Delayed conidial germination of *M. robertsii* and *B. bassiana* was also reported on the cuticle of *D. nitens* and *A. sculptum* using scanning electron microscopy. The toxicity of cuticular extracts from *A. sculptum* or *D. nitens* to *M. robertsii* and *B. bassiana* was determined by conidial death. Additionally, this study characterized the cuticular neutral lipids and hydrocarbons of these ixodid ticks treated or not treated with entomopathogenic fungi.

## 3. Conclusions

We highlight that these Special Issues contain twelve articles from eight countries from South and North America, Asia, and Africa: three articles from Brazil, two from Argentina and the USA, and one from Mexico, Egypt, Lebanon, China, and South Africa. These data reinforce the importance of producing comprehensive research on entomopathogenic fungi, which aligns with the global demand for eco-friendly alternatives for pest management to minimize the impact of insect pests and arthropod-borne diseases on world health and economies. 
